# The impact of China’s non-pharmaceutical interventions on economic growth during COVID-19: a provincial-level panel analysis

**DOI:** 10.1186/s12889-026-28461-3

**Published:** 2026-07-08

**Authors:** Yisha Ma, Barjoyai Bin Bardai

**Affiliations:** 1 Xian Kedagaoxin University, Xi’an, Shaanxi Province China; 2 Faculty of Management, Malaysia University of Science and Technology, Petaling Jaya, Selangor Malaysia

**Keywords:** COVID-19, Non-pharmaceutical interventions (NPIs), Economic impact, China, Regional heterogeneity, Dynamic model

## Abstract

This study examines the economic impacts of non-pharmaceutical interventions implemented in China during the COVID-19 pandemic, utilizing a provincial-level panel dataset from 2020Q1 to 2022Q4. We employ a pooled OLS model with panel-corrected standard errors (PCSE) to address cross-sectional dependence, serial correlation, and heteroskedasticity, while a fixed-effects instrumental variable (FE-IV) model is used to resolve endogeneity from reverse causality. The analysis reveals that NPIs significantly reduced quarterly GDP growth, with coefficients ranging from -0.078 to 0.154 across models. Regional heterogeneity shows stronger negative effects in eastern (-0.092) provinces compared to western and central provinces. Temporal analysis further identifies an empirically observed three-stage targeted response: significant negative impacts in 2020 (-0.180), but an insignificant effect in 2021 and 2022, reflecting policy adjustments and economic adaptation. The findings underscore the trade-offs between public health measures and economic performance, with an estimated 0.096 percentage point economic loss due to NPIs, consistent with global studies.

## Introduction

The global spread of the COVID-19 pandemic was gradual [20]. To control the virus, the city of Wuhan announced a citywide closure policy on January 23, 2020 [42]. The economic impacts were severe, as productive activities were halted on a large scale [53]. Meanwhile, 230 countries and regions worldwide^1^ were affected.^2^At the onset of the pandemic, countries^3^ implemented NPIs,^4^which slowed the spread of COVID-19 [45], which in turn led to a massive economic downturn [22]. There is growing evidence of the negative impacts of epidemics on morbidity [56] and mortality [8]. However, the damage caused by NPIs [58] far exceeds the direct impacts of the virus itself [25, 29]. Briones et al. [14] estimated the economic impacts of border closures and social distancing by analyzing GDP data for Kenya, Singapore, and Thailand. Fairlie [27] conducted an analysis using the microdata from the U.S. Current Population Survey (CPS) covering February to April 2020. The findings revealed that social isolation measures during the pandemic led to a 22% decline in active micro and small business ownership, impacting approximately 3.7 million businesses in the United States.

While NPIs have been widely adopted globally, China’s implementation of NPIs during the COVID-19 pandemic was significantly different from that of most countries globally, making research focusing on China uniquely valuable. Unlike many countries that shifted from stringent NPIs in the later phase of the pandemic to adopting a living-with-the-virus policy [21, 47] in the later stage, China adhered firmly to the core strategy of “Dynamic Zero-COVID” from 2020 to 2022 [6, 19, 52, 54]. It enforced NPIs, including city-wide lockdowns, universal contact tracing, strict mobility controls, and mandatory quarantine for close contacts. These policies were characterized by long duration, strong enforcement, and a consistent goal of virus suppression rather than mitigation. This persistent commitment to a Dynamic zero-COVID strategy has provided a unique empirical context to systematically investigate the long-term economic costs of sustained NPIs, addressing an important gap in the literature that has largely centered on short-term lockdowns.

This paper introduces significant innovations across data, methodology, empirical findings, policy quantification, and theoretical framework. First, in terms of data, it is the first to systematically evaluate the dynamic effects of NPIs on economic growth using quarterly provincial panel data from China (2020Q1–2022Q4). Compared to existing studies that mostly rely on cross-country or national-level data, the use of higher-granularity provincial data allows for a more precise capture of regional heterogeneity, filling the gap in “real-time, localized” empirical evidence.

Second, methodologically, the paper adopts a sequential estimation strategy: we first use pooled OLS and fixed effects (FE) as exploratory models, then select the corrected standard errors (PCSE) to address cross-sectional dependence, serial correlation, and heteroskedasticity in provincial panel data. To resolve endogeneity, a fixed-effects instrumental variable (FE-IV) model is further employed. Rigorous unit root tests, and heteroskedasticity/autocorrelation diagnostics are conducted to ensure robustness. Particularly, by introducing the PCSE, the study effectively addresses cross-sectional dependence and serial correlation in provincial data, further enhancing the reliability of the estimates.

In terms of empirical findings, the paper uncovers notable spatiotemporal heterogeneity in the effects of NPIs. Spatially, the economic impact is significantly larger in the eastern (−0.092) regions compared to the western region and central, highlighting how regional economic structures influence policy sensitivity. Temporally, the study is the first to propose a “three-stage targeted response” of NPIs: a significant negative impact in 2020 (−0.180), an insignificant effect in 2021 (0.034, reflecting policy adjustment and economic adaptation), and an insignificant negative impact again in 2022 (−0.012), providing a time-based rationale for flexible policy adjustments.

Moreover, regarding policy quantification, the paper empirically estimates that NPIs led to approximately an 0.096 percentage point economic loss in China, comparable to global studies (such as 0.09 estimated by [39]. For the first time, this figure is validated within the context of China’s prolonged strict containment strategy, offering critical data support for evaluating the cost–benefit of the “zero-COVID” policy.

Most importantly, in terms of theoretical contribution, this study moves beyond the prevailing static analysis of NPIs' economic costs. Building upon and synthesizing established theories of economic resilience, adaptation to shocks, and policy learning curves, we derive an empirically observed "three-stage dynamic model" of the public health–economy trade-off. This empirically observed pattern is characterized by three phases: (1) the emergency control phase (2020), where stringent, uniform NPIs dominate, leading to significant economic contraction; (2) the balanced adaptation phase (2021), where economic agents and policymakers learn and adapt, mitigating the marginal cost of interventions through behavioral changes and targeted support; and (3) the post-vaccine phase (2022), where the resurgence of NPIs amidst a changed macro-environment (e.g., waning stimulus, external pressures) reveals renewed economic resilience.

The value of three-stage targeted response pattern lies not in asserting that NPIs impose costs, a well-established finding, but in providing a testable, mechanism-based explanation for how and why these costs evolve non-linearly over the lifecycle of a prolonged crisis. China’s sustained “Dynamic Zero-COVID” strategy serves as a critical empirical setting, a rigorous “stress test”, to validate this observed pattern using high-frequency subnational data. Thus, this observed pattern offers a systematic tool for understanding the dynamic relationship between containment measures and economic performance, offering nuanced insights for adaptive policy adjustment in future pandemics.

The structure of this paper is organized as follows: Sect. " Literature review" reviews the literature on historical pandemics, NPIs, their economic impacts, and the theoretical mechanism. Sect. " Methodology" details the data, econometric models (pooled OLS, PCSE, FE-IV), and data analysis procedures. Sect. " Results" reports the baseline results, area/year heterogeneity, and robustness checks. Sect. " Discussion" discusses the health-economic trade-off, derives policy implications, and addresses limitations and future work. Sect. "Conclusion" concludes the study.

## Literature review

### Historical pandemics and economic growth

From ancient times, humanity has encountered countless pandemic, some of which have been particularly serious. Some of these pandemics have been particularly serious and have significantly impacted the survival and development of humanity, for example, the bubonic plague, smallpox, influenza, cholera, malaria, and so on. Supplementary Table A1 shows the huge amount of loss in human lives and the economy caused by epithet pandemics. According to [8], 6% to 8% of GDP was lost as a result of the 1918 influenza epidemic. Carillo and Jappelli [16] evaluated how the 1918 influenza affected Italy's economy and found that the country's GDP per capita growth rate had dropped by 6.5 percent. Brainerd and Siegler [13] analysed data for the United States from 1919 to 1930 and concluded that influenza would positively influence the country’s per capita income growth over the ensuing decade. The Asian flu killed one to two million people in 1957 and vanished a year later, and caused an economic loss of 0.58% of GDP in the United Kingdom (UK) [35]. Drawing on historical evidence, Bloom and Cadarette [11] documented that over the past century, repeated pandemics have been accompanied by large numbers of cases and high mortality rates, reinforcing the severe demographic and economic consequences illustrated in supplementary Table A1. SARS, H1N1 and Ebola outbreaks have devastated many countries, population and economy, as shown in supplementary Table A1.

### An overview of Non-Pharmaceutical Interventions (NPIs)

Since modern times, during the 1918 Spanish Flu pandemic, many countries implemented measures such as restricting public gatherings and closing schools, which laid the foundation for modern NPIs [18]. Although these historical practices were relatively simplistic, they already embodied core logic including isolation, mobility restrictions, and environmental disinfection, providing valuable experience for subsequent epidemic prevention efforts [33].

NPIs refer to a general term for various public health strategies that prevent and control disease transmission by adjusting human behaviour, optimizing environmental conditions, or strengthening public management, without relying on drugs or vaccines. Their core goal is to reduce the risk of virus exposure and alleviate the pressure on medical systems. Specifically, these measures include patient isolation, contact tracing, quarantine of close contacts, travel restrictions, closure of schools and workplaces, cancellation of mass gatherings, and hand hygiene promotion. By delaying the timing and magnitude of the COVID-19 peak to buy time for medical system preparedness, transportation interdiction and community-level NPIs have been proven to be among the most effective tools.

During the COVID-19 pandemic, the rapid development of information technology has promoted the systematization and precision of NPIs, and also spawned several globally unified quantitative databases for NPIs [25]. Among them, the Oxford COVID-19 Government Response Tracker (OxCGRT) is the most widely used [31]. This database generates quantitative indicators such as the Government Response Stringency Index by tracking 20 policy indicators, providing a standardized tool for cross-country and cross-regional comparisons of NPI effectiveness. This paper adopts the core indicators of the OxCGRT database to ensure the comparability and reliability of the research results. Detailed data content and relevant descriptive statistics are presented in supplementary appendix B.

### Empirical studies on the economic impacts of COVID-19 NPIs

It is evident through studies such as [1, 5, 24, 28, 32, 51] that these papers employ data from the OxCGRT to analyze economic issues. Furceri et al. [28] used Ordinary Least Squares (OLS) and Weighted Average Least Squares (WALS) to elaborate on the relationship between NPIs from the OxCGRT and economic output. König and Winkler [38] used economic data for 42 countries and obtained the result that mortality plays an important role, but a panel regression showed that embargo stringency is a more important driver of growth and the regression coefficient in the first quarter of 2020 was −17 percentage point. An analysis of data from 44 countries, argued that for every 1% increase in the stringency index of NPIs, the economy would fall by 9 percentage point [39].

Unlike cross-country studies that focus on short-term lockdowns [10, 26, 30], this study’s identification of a “three-stage dynamic balance” is unique to China’s sustained “Dynamic Zero-COVID” strategy. Most countries abandoned strict containment prematurely or oscillated between loose and strict measures, but China’s long-term commitment to virus suppression allowed for the observation of how the health-economy trade-off evolves with policy persistence, adaptation, and external shocks. This supplements global literature’s gap in empirical evidence on long-term NPIs’ economic impacts, enriching the contextual diversity of health-economy trade-off research.

Prior Chinese studies documented economic disruptions from SARS [50] and early COVID-19 lockdowns [40, 41], but lacked systematic analysis of spatiotemporal heterogeneity and dynamic balance. This study supplements this by quantifying regional differences in NPIs’ economic impacts and linking temporal variations to the evolving health-economy trade-off. Using provincial panel data also captures localized variations in policy implementation and economic resilience, providing more granular evidence for region-specific policy design. This offers an advantage over national-level analyses.

Few studies [24, 55] have conducted empirical evaluations using provincial-level panel data to examine the real-time, dynamic impact of NPIs on regional GDP growth. This paper addresses this gap by employing a panel regression model on quarterly data from 30 Chinese provinces between 2020 and 2022, providing updated and localized evidence on the economic consequences of pandemic response policies. Caporale et al. [15] employed Generalized Method of Moments (GMM) and Panel-Corrected Standard Errors (PCSE) approaches to analyze longer-span quarterly data (2000Q1–2021Q4) for European countries, finding that the COVID-19 pandemic exerted significant negative impacts on both exports and imports in the short and long run.

#### Hypothesis

There is a significant relationship between NPIs and GDP.

### Theoretical framework and mechanism

The existing literature confirms that NPIs reduce virus transmission but generate short-term economic costs by disrupting production, consumption, labor mobility, and supply chains. However, most studies adopt a static perspective and fail to explain how the economic impact of NPIs evolves over time under prolonged and consistent policy enforcement. This study establishes a unified theoretical framework to clarify the transmission mechanism through which NPIs affect regional economic growth.

#### Theoretical motivation

The core theoretical logic of this study is the dynamic trade-off between public health protection and economic activity. During public health crises, stringent NPIs effectively reduce infection risks but constrain economic behavior. As government adapt and policies become more targeted, the marginal economic cost of NPIs gradually changes. This study focuses on the spatiotemporal heterogeneity of this trade-off using subnational panel data, which allows more precise identification than aggregate national or cross-country analyses.

This study’s theoretical contribution lies in two aspects: First, it breaks through the static perspective of existing studies and constructs a dynamic theoretical framework that incorporates the adaptive behavior of economic agents, which solves the theoretical puzzle that existing studies cannot explain the dynamic change of economic effects under long-term NPIs. Second, by integrating the regional heterogeneity of China’s Dynamic Zero-COVID strategy into the theoretical framework, this study supplements the theoretical gap of sub-national level NPI economic effect research, which is more targeted than cross-country theoretical studies. This addresses the concern that the core research problem is trivial by highlighting the unique value-added of this study.

#### Transmission mechanism of NPIs on economic growth

NPIs influence economic growth through three parallel channels:​

Supply-side disruption: Mobility restrictions (especially C7 restrictions on internal movement), workplace closures (C2), and production suspensions reduce labour input, interrupt logistics, and suppress output in industry and services. Specifically, higher NPI intensity leads to stricter restrictions on labour mobility, which further reduces industrial output and ultimately slows down GDP growth.​

Demand-side contraction: Gathering bans (C3), travel restrictions (C7, C8), and risk aversion reduce offline consumption, tourism, and catering services, weakening aggregate demand. This channel directly links NPI stringency to the decline of aggregate demand, thereby negatively affecting regional GDP growth.​

Adaptive mitigation: Over time, firms and households adjust behaviour (e.g., remote work, digital consumption, flexible operations). Governments also shift from blanket restrictions to targeted interventions (e.g., implementing measures only in medium–high risk areas). These adaptations reduce the marginal economic impact of NPIs, and this mitigation effect strengthens over time, which weakens the negative impacts of the supply-side disruption and demand-side contraction channels [46].

Notably, regional economic resilience plays a crucial role in the adaptive mitigation channel. Economic resilience, defined as the ability of a region to resist, adapt to, and recover from the economic shocks caused by NPIs, directly determines the effectiveness of adaptive behaviors of economic agents. Regions with higher economic resilience can better absorb the negative impacts of NPIs through industrial restructuring, technological innovation, and policy adaptation, thereby reducing the decline in GDP growth caused by NPIs.

## Methodology

This study employs a quarterly provincial panel dataset covering 30 Chinese provinces from 2020Q1 to 2022Q4. Data are collected from the National Bureau of Statistics (NBS), CEIC, and the OxCGRT provincial policy database [59]. A sequence of panel data methods is applied to identify the causal effect of NPI stringency on economic growth.

### Data collection

In this study, to verify the impact of COVID-19-related indicators on the economic growth of Chinese provinces, pooled OLS (ordinary least squares) regression analyses. The results show that the bivariate scatter plots of provincial quarterly GDP growth with NPI intensity and pandemic outcome variables, providing preliminary evidence on the simple correlations between key variables. NPI intensity exhibits a stable and significantly negative linear correlation with gdpgrow, with no severe outlier interference. In contrast, new COVID-19 cases and deaths are highly right-skewed, with most observations clustering at zero and only a few extreme values driving the negative correlation. Therefore, in this paper, the stringency of NPIs is chosen as the representative of COVID-19 as the independent variable [4]. The variable of new cases is used as a control variables in the regression [17], while new_deaths is temporarily excluded from subsequent regression specifications. We retain only new_cases as a control variable to capture quarterly pandemic severity in a parsimonious and interpretable manner. The dependent variable was selected based on previous studies [36, 37, 39, 43] and is defined as quarterly GDP growth.

### Model specifications

This section formally presents the econometric specifications for pooled OLS, two-way fixed effects (FE), pooled OLS with panel-corrected standard errors (PCSE), and fixed-effects instrumental variable (FE-IV). For each method, we specify the estimation equation, assumed error structure, and key identifying assumptions.

#### Pooled OLS model

1 $${\mathrm{G}\mathrm{d}\mathrm{p}\mathrm{g}\mathrm{r}\mathrm{o}\mathrm{w}}_{it}={\alpha}_{0}+{\alpha}_{1}{NPI}_{it}+{\alpha}_{2}{C}_{it}+{\varepsilon}_{it}$$

${\varepsilon}_{it}$ i.i.d., homoskedastic, no cross-sectional dependence, no serial correlation. No unobserved provincial or time heterogeneity; NPI is uncorrelated with ${\varepsilon}_{it}$​ (no endogeneity).

#### Two-Way Fixed Effects (FE) model

2 $${\mathrm{G}\mathrm{d}\mathrm{p}\mathrm{g}\mathrm{r}\mathrm{o}\mathrm{w}}_{it}={\alpha}_{0}+{\alpha}_{1}{NPI}_{it}+{\alpha}_{2}{C}_{it}+{\mu}_{\mathfrak{i}}+{\lambda}_{t}+{\varepsilon}_{it}$$

${\varepsilon}_{it}$ heteroskedastic, no serial correlation, no cross-sectional dependence. Standard errors clustered at the provincial level. ${\mu}_{\mathfrak{i}}$ (Provincial FE) and ${\lambda}_{t}$​ (time FE) capture unobserved heterogeneity; NPI is uncorrelated with ${\varepsilon}_{it}$ (no endogeneity).

#### Pooled OLS with Panel-Corrected Standard Errors (PCSE)

PCSE is not an independent estimator but a standard-error correction applied to pooled OLS [9]. Coefficients are identical to pooled OLS, only standard errors are adjusted.

3 $${\mathrm{G}\mathrm{d}\mathrm{p}\mathrm{g}\mathrm{r}\mathrm{o}\mathrm{w}}_{it}={\alpha}_{0}+{\alpha}_{1}{NPI}_{it}+{\alpha}_{2}{C}_{it}+{\varepsilon}_{it}$$

$\mathcal{i}$ (1, …,30) denotes provinces, and t (1, …,12) denotes quarters;

${Gdpgrow}_{it}$-The quarterly GDP growth rate for each province in China is a dependent. variable of the quarterly GDP growth rate for each province in China.

${NPI}_{it}$-core explanatory variable, independent variable, the number of the quarterly. stringency of NPIs for each province in China.

${C}_{it}$- the control variables:

New_case: ${confirm\_cases}_{it}-{confirm\_cases}_{it-1}$. This calculation ensures that new_cases captures the flow of newly confirmed cases in each quarter, aligning with the flow nature of the dependent variable gdpgrow(quarterly GDP growth rate) and avoiding stock-flow mismatch.

InGexpenditure: Logarithm of quarterly provincial government general budgetary. expenditure (100 million yuan);

IVA: Quarterly industrial value-added growth rate;

Import_export: Total value of imports and exports, represented by its accumulated. year-on-year growth rate (%).

$${\alpha}_{0}$$-Constant.

${\alpha}_{1}$- the coefficient associated with the variable "NPI."

${\mu}_{\mathfrak{i}}$- the individual fixed effect, capturing time-invariant provincial heterogeneity (e.g., geographical location, industrial structure);

${\lambda}_{t}$-the time fixed effect, accounting for common temporal shocks (e.g., national pandemic policies, global economic fluctuations);

$${\varepsilon}_{it}$$- idiosyncratic error.

#### Panel-Corrected Standard Errors (PCSE)

While the two-way FE model controls for individual and time heterogeneity, it fails to address three fundamental challenges in macro-panel data (detailed diagnostic results reported in Sect. " Diagnostic tests for panel data properties" and (Table E2 Justification for Using the PCSE Model):

1. Heteroskedasticity: Modified Wald test (χ^2^ = 2438.19, *p* < 0.001) confirms group-wise heteroskedasticity across provinces;
2. Serial Correlation: Wooldridge test (F = 59.999, *p* < 0.001) indicates significant first-order serial correlation within provinces;
3. Cross-Sectional Dependence: Pesaran CD test (CD = 28.69, *p* < 0.001) rejects cross-sectional independence, reflecting common shocks across provinces (e.g., national epidemic prevention policies).

Diagnostic tests were conducted to address potential issues in panel data (Table E2). Importantly, PCSE is not an independent estimation model but a standard-error correction applied to pooled OLS [9]. It retains OLS coefficient estimates while adjusting standard errors for heteroskedasticity, serial correlation, and cross-sectional dependence, common challenges in macro-panel data analysis. The core mathematical principle of PCSE is adjusting the variance–covariance matrix to address heteroskedasticity, serial correlation, and cross-sectional dependence:

4 $${\widehat{\Omega }}_{PCSE} ={({X}^{^{\prime}}\mathrm{X})}^{-1}{X}^{^{\prime}}\widehat{\sum }X{({X}^{^{\prime}}\mathrm{X})}^{-1}$$

where:

X—the matrix of explanatory variables (including constants, NPI_it_, C_it_, ${\mu}_{\mathfrak{i}} {\lambda}_{\mathfrak{i}}$);

$\widehat{\sum }$—the estimated covariance matrix of errors, corrected for cross-provincial variance differences, first-order serial correlation, and contemporaneous cross-sectional correlation.

Our baseline specification is pooled OLS, with PCSE employed as a standard‑error correction. Given that PCSE may be less reliable in our short panel (T < N), we further conduct robustness checks using Driscoll–Kraay standard errors, FGLS, and bootstrap‑based inference.

#### Fixed-Effects Instrumental Variable (FE-IV) Model

To address potential endogeneity arising from reverse causality between NPIs and economic growth, a Fixed-Effects Instrumental Variable (FE-IV) model [3] is further employed as a robustness check to address endogeneity. Following the two-stage least squares (2SLS) framework, we use the one-period lag of NPI (L.NPI_it_) as the instrumental variable, which satisfies two key validity criteria:

1. Relevance: The Wald F-statistic (59.999) exceeds the critical value of 10, confirming a strong correlation with the endogenous variable NPI_it_ and ruling out weak instrument bias;
2. Exogeneity: The lagged NPI is temporally predetermined and unaffected by current economic shocks. A supplementary Panel Vector Autoregression (PVAR) Granger causality test further verifies that Gdpgrow_it_ has no significant reverse impact on L.NPI_it_, supporting the exclusion restriction. Detailed results of the PVAR analysis are presented in supplementary appendix G.

The two-stage specification of the FE-IV model is as follows:

- Stage 1: Predict the endogenous variable (NPI_it_).

We regress the endogenous core explanatory variable (NPI_it_) on the instrumental variable (L.NPI_it_) and all control variables, while retaining individual and time fixed effects to control for unobserved heterogeneity:

5 $${\mathrm{N}\mathrm{P}\mathrm{I}}_{it}={\delta}_{0}+{\delta}_{1}L.{NPI}_{it}+{\delta}_{2}{C}_{it}+{\mu}_{\mathfrak{i}}+{\lambda}_{t}+{\nu}_{it}$$

where:

$L.{NPI}_{it}$—the one-period lag of ${NPI}_{it}$ (instrumental variable);

${C}_{it}$- the control variables, such as new_case, InGexpenditure, IVA, import_export.

${\nu}_{it}$—error term of the first-stage regression;

All other variables retain the same definition as in Eq. (1).

- Stage 2: Estimate the casual effect.

Replace the endogenous variable (NPI_it_) with its predicted value (${\widehat{NPI}}_{it}$) from the first stage, then regress it on the dependent variable to obtain the unbiased causal effect of NPI on economic growth:

6 $${\mathrm{G}\mathrm{d}\mathrm{p}\mathrm{g}\mathrm{r}\mathrm{o}\mathrm{w}}_{it}={\alpha}_{0}+{\alpha}_{1}{\widehat{NPI}}_{it}+{\alpha}_{2}{C}_{it}+{\mu}_{\mathfrak{i}}+{\lambda}_{t}+{\varepsilon}_{it}$$

where:

• $\widehat{NPI}_{it}$— predicted value of ${NPI}_{it}$ from Stage 1 (purified of endogeneity);

• ${C}_{it}$— the control variables, such as new_case, InGexpenditure, IVA, import_export.

• ${\varepsilon}_{it}$— error term of the second-stage regression;

• ${\alpha}_{1}$— the causal effect of NPI stringency on GDP growth (our key parameter of interest);

• All other variables retain the same definition as in Eq. (3).

### Data analysis

This paper collects the COVID-19 pandemic and economic data for 30 provinces in China^5^ from 2020Q1-2022Q4.^6^It includes quarterly COVID-19 data, such as the new number of cases and the stringency of NPIs. In this paper, during the research sample, data are sourced from the China Statistics Network, CEIC, OxCGRT, etc. (see Table D1 Variables descriptions and Table D2 The detail of the way to measure variables in the supplementary appendix D). The following data analysis proceeds in five steps to justify the final model selection:

#### Descriptive statistics

Table 1 presents the descriptive statistics of core variables (N = 360). The quarterly GDP growth rate (gdpgrow) has a mean of 4.71% and with a large fluctuation range from −39.30% to 58.30%, reflecting the overall stable positive growth of provincial economies during 2020Q1–2022Q4. The minimum value of −39.3% (a minor discrepancy from the official figure of −39.2%^7^ due to statistical rounding, with the data in this study prevailing) corresponds to Hubei Province’s GDP growth in 2020Q1, a direct impact of the initial COVID-19 pandemic and NPIs, specifically the city-wide lockdown of Wuhan (implemented on January 23, 2020) and subsequent mobility restrictions across Hubei. This unprecedented lockdown halted most productive and economic activities, leading to a sharp economic contraction. In contrast, the maximum growth rate of 58.3% is Hubei’s GDP growth in 2021Q1, indicating a strong post-pandemic economic rebound driven by the resumption of production, policy stimulus, and pent-up demand.

**Table 1:** Descriptive statistics

Variable	Obser vations	Mean	Std. Dev	Min	Max
GDP growth rate (gdpgrow)	360	4.71	7.12	−39.3	58.3
NPI stringency index (NPI)	360	49.88	10.18	19.89	79.2
Industrial value-added growth (iva)	360	5.59	8.92	−46.9	90.3
Quarterly new COVID-19 cases (new_cases)	360	917.6	3831.7	0	31,799
Government expenditure (InGexpenditure)	360	7.88	0.87	5.41	9.72
Import & export growth rate (import_export)	360	14.37	24.33	−63.73	106.17
Tertiary industry growth (tertiaryindustry)	360	4.51	6.53	−33.3	45.7

For the NPI stringency index, the mean (49.88) suggests moderately stringent policies across most provinces, with a range of 19.89–79.2 capturing significant cross-provincial policy differences, critical for identifying NPI’s economic impact. The industrial value-added growth rate (IVA) ranges from −46.9% to 90.3%, with the minimum (−46.9%) and maximum (90.3%) both corresponding to Hubei Province in 2020Q1 and 2021Q1, respectively. The 2020Q1 slump (−46.9%) stems from factory shutdowns and supply chain disruptions caused by Hubei’s lockdown, while the 2021Q1 surge (90.3%) reflects the robust recovery of manufacturing and industrial sectors as restrictions were lifted.

Quarterly new COVID-19 cases (new_cases) are constructed by using the end-of-quarter cumulative confirmed cases. Quarterly new COVID-19 cases (new_cases) exhibit a highly right-skewed distribution, with a mean of 917.60, a standard deviation of 3831.70, and a maximum of 31,799. The large gap between the mean and median indicates that most provincial-quarters recorded zero or a small number of new cases, while a few pandemic outbreaks (e.g., concentrated infections in populous provinces) drove up the average, consistent with the “local clusters and national sporadic” pandemic pattern during the sample period. Quarterly new COVID-19 deaths (new_deaths) are used in exploratory analyses but excluded from the main regression models. This effect contest undermines the interpretability of pandemic-related shocks. Since the core objective of this study is to identify the causal effect of NPI stringency on economic growth, we retain only new_cases as a control variable to capture quarterly pandemic severity in a parsimonious and interpretable manner.

The relatively concentrated government expenditure (InGexpenditure,5.41–9.72) reflects reasonable cross-provincial differences in policy intervention intensity. Import & export growth rate (import_export) is measured as the accumulated year-on-year growth rate of total imports and exports. This variable has 360 observations, with a mean value of 14.37, a standard deviation of 24.33, and ranges from a minimum of −63.73 to a maximum of 106.17. The variable is included to capture external demand shocks and export-led economic fluctuations, thereby addressing potential omitted variable bias related to global demand conditions during the pandemic. The tertiary industry growth rate (tertiaryindustry) has a mean of 4.51% and a standard deviation of 6.53, with a range from −33.3% to 45.7%. As a key component of the provincial economy, tertiary industry (e.g., services, retail, and tourism) is highly sensitive to pandemic restrictions and mobility controls. The negative minimum value (−33.3%) reflects the severe impact of lockdown measures on contact-intensive service sectors in 2020Q1, while the positive maximum value (45.7%) indicates a strong recovery in tertiary industry activities as pandemic controls eased. This further validates the robustness of our baseline findings when using tertiary industry growth as an alternative measure of economic performance.

#### Correlation and multicollinearity tests

First conduct pairwise correlation analysis to initially verify the theoretical associations between variables (NPIs, GDP growth, and control variables) proposed in this section, which helps exclude severe multicollinearity and provides preliminary evidence for the theoretical mechanism, laying a foundation for subsequent model setting. The main variables were analyzed for correlation using the Pwcorr correlation test, and the results are presented in Table 2. It can be seen that the correlation coefficients between the new number of cases and deaths, and the average stringency of NPIs, are related to the COVID-19 pandemic variable selected in this paper. The economic growth rates are −0.011 and −0.970***, respectively, initially supporting this paper's research hypothesis. This finding is consistent with other analytical results in the paper. For example, the baseline regression results show that an increase in the stringency of NPIs leads to a decline in economic growth. In addition, the result of the VIF variance inflation factor has success in the subsequent model regressions. The VIF value (1.11) is much less than 10, indicating no serious multicollinearity among the variables.

**Table 2:** Pairwise correlations

Variables	(1)	(2)	(3)	(4)	(5)	(6)	(7)	(8)
(1) gdpgrow	1.000							
(2) NPI	−0.258***	1.000						
(3) IVA	0.589***	−0.162***	1.000					
(4) new_cases	−0.279***	0.269***	−0.364***	1.000				
(5) InGexpenditure	−0.011	0.019	−0.051	0.064	1.000			
(6) import_export	0.546***	−0.138***	0.196***	−0.086*	0.008	1.000		
(7) tertiaryindustry	0.970***	−0.300***	0.495***	−0.262***	−0.019	0.570***	−0.411***	1.000

^***^ p < 0.01, ** p < 0.05, * p < *0.1*

#### Unit root tests

To avoid spurious regression, we first test for cross-sectional dependence (CD) using the Pesaran CD test. Significant CD is confirmed, so we proceed with second-generation panel unit root tests (CIPS, CADF) alongside first-generation tests (LLC, IPS, Fisher-ADF, Fisher-PP, HT). The combined results of the eight panel unit root tests (indicate detailed in supplementary appendix E Table E1) reveal that most core and control variables (NPI, iva, new_cases, import_export, InGexpenditure), the p-values of key unit root tests (LLC, IPS, HT, Fisher-PP) are all less than 0.05, indicating strong statistical significance and confirming their stationarity.^8^For two variables, InGexpenditure and gdpgrow, the test results show "stationary (near critical)": InGexpenditure has a Pesaran CIPS value of −2.331 (close to the 5% critical value of −2.17), and gdpgrow has a Pesaran CIPS value of −2.161 (slightly higher than the 5% critical value of −2.17); however, the LLC and IPS tests for both variables still yield significant p-values (p < 0.05), which, combined with the overall test results, sufficiently support their stationarity. Similarly, the remaining variables is confirmed to be stationary, as its LLC and IPS test p-values are less than 0.05, and the Pesaran CIPS value is close to the critical value, with the Fisher-PP test also showing significant results.

Therefore, we directly use the level values of all variables for regression analysis, which can yield unbiased and consistent estimates. This approach not only retains the level information and long-run trend characteristics of each variable but also aligns with the core research objective of this paper, to explore the long-run impact of NPI on economic growth. The stationary nature of all variables allows us to directly interpret the coefficients as long-run marginal effects, ensuring the economic significance and reliability of the regression results.

#### Diagnostic tests for panel data properties

Taken together, the diagnostic results on heteroskedasticity, serial correlation, cross-sectional dependence, and fixed effects strongly justify the use of PCSE (Table E2), which corrects standard errors while preserving province-specific effects. PCSE provides efficient inference by correcting for complex panel data structures while preserving province-specific effects, yielding a well-fitting model (R^2^ = 0.671) and a stable coefficient estimate for our primary analysis.

However, to credibly claim a causal effect of NPIs on economic growth, we must confront a key identification challenge: the potential for simultaneous determination. The significant Durbin-Wu-Hausman (DWH) test (χ^2^ = 13.350, p = 0.0003) in Table E2 identifies significant endogeneity, suggesting potential reverse causality where economic performance could influence policy stringency, necessitating a robust causal identification strategy, this does not invalidate the application of PCSE for baseline inference.

#### Rationale for final model selection

It establishes a clear rationale for employing Fixed-Effects Instrumental Variable (FE-IV) estimation. Our approach addresses this challenge in two key steps. First, we establish the need for an instrument: the significant DWH test confirms potential bias in standard estimators. Second, we rigorously justify our instrument choice: we use the one-period lag of NPI (L.NPI), which is strongly correlated with the endogenous variable (Wald F-statistic = 51.474). Critically, the use of this temporally predetermined instrument is empirically scrutinized and informed by a supplementary Panel Vector Autoregression (PVAR) analysis (supplementary appendix G). The analysis reveals a statistically significant bidirectional Granger-causality, with the feedback from economic growth to NPI being negative in sign. By employing L.NPI, a temporally predetermined variable, we isolate policy variation that is exogenous to concurrent economic shocks, thereby yielding a more credible estimate of the causal effect of NPIs on economic growth.

Despite the short-panel nature of our data (N = 30, T = 12; T < N), we follow established methodological literature and adopt pooled OLS with PCSE correction. Applied studies confirm that PCSE remains reliable for panels with moderate N (≤ 30), strong cross-sectional dependence, and serial correlation, even when T is small [2, 7, 34]. In such settings, PCSE outperforms conventional cluster-robust standard errors in finite samples, making it suitable for our provincial micro-panel.

In summary, pooled OLS with PCSE yields a better model fit and a coefficient magnitude (approximately 0.096), fulfilling the needs of “province-specific” regressions. Therefore, PCSE serves as the main estimation strategy to account for cross-sectional dependence, heteroskedasticity and panel-specific first-order autocorrelation (AR(1)) simultaneously, while FE-IV models supplement the analysis to address potential endogeneity concerns and verify the causal robustness of the core findings.

## Results

### Baseline regression results

This paper takes the pooled OLS model as our model, with PCSE. Meanwhile, FE, and FE-IV models as supplements to verify result robustness. Definitions of each model are as follows:

- Pooled OLS: pooled OLS regression serves only as a correlational benchmark and does not imply causal inference; formal estimation relies on models that control for individual and time effects."
- FE: Fixed effects model with cluster-robust standard errors, controlling for unobserved provincial heterogeneity and mitigating potential biases from time trends embedded in control variables (e.g., accumulated fiscal expenditure and industrial value added) through time fixed effects.;
- FE-IV: Fixed effects instrumental variables regression (using lagged NPI as instrument), employed as a robustness check to address endogeneity;
-PCSE-PSAR1: Panel-corrected standard errors model with AR(1) serial correlation assumption, serving as the core model to correct for cross-sectional dependence, serial correlation, and heteroskedasticity.

The key independent variable, NPI, consistently shows a negative and significant impact on GDP growth across all models. The estimated coefficients are −0.078 (1), *p* < 0.01), −0.154 (FE-IV, *p* < 0.01), −0.177 (FE, p < 0.01), and −0.096 (PCSE-PSAR1, *p* < 0.05) (Table 3). As our preferred model, the PCSE-PSAR1’s coefficient (−0.096) has a straightforward economic interpretation: a one-unit increase in NPI stringency is associated with a 0.096 percentage point decline in quarterly GDP growth. This indicates that an increase in NPI stringency is associated with a decline in GDP growth, with the magnitude of impact ranging from 0.078 to 0.154 percentage point depending on the model. Notably, the FE-IV model yields the largest negative coefficient (−0.154), suggesting that correcting for endogeneity amplifies the estimated impact of NPIs. In contrast, the PCSE-PSAR1 model produces a smaller effect size, potentially because it accounts for serial correlation and province-level heterogeneity.

**Table 3:** Regression results for GDP growth

Variables	(1) Pooled OLS	(2) PCSE-PSAR1	(3) FE	(4) FE-IV
NPI	–0.078*** (0.026)	–0.096** (0.046)	–0.177*** (0.033)	–0.154*** (0.028)
iva	0.377*** (0.031)	0.359*** (0.050)	0.375*** (0.095)	0.299*** (0.105)
new_cases	–0.000 (0.000)	–0.000 (0.000)	–0.000 (0.000)	–0.000 (0.000)
InGexpenditure	0.121 (0.290)	–1.383 (0.850)	–1.513*** (0.245)	–2.764*** (0.339)
import_export	0.127*** (0.011)	0.129*** (0.013)	0.103*** (0.025)	0.099*** (0.025)
_cons	3.769* (2.658)	—	—	—
t	—	0.592** (0.230)	0.583*** (0.088)	0.301*** (0.088)
F/Wald χ^2^	F = 88.22***	χ^2^ = 89,525.66***	F = 17.85***	χ^2^ = 10,722.47***
First-stage F	—	—	—	51.474
Hansen J p-value	—	—	—	0 (exact ID)
Individual FE	No	No	Yes	Yes
Cluster SE	No	PCSE	Yes	Yes
Observations	360	360	360	330
R-squared	0.555	0.671	0.611	0.562

Full province-level coefficient estimates are reported in supplementary appendix F Table F1

Among the control variables, the time trend (t) is positively and significantly associated with GDP growth, suggesting a steady upward trend in GDP growth over time, likely reflecting ongoing structural economic improvements or technological progress. Other controls also show expected signs: industrial value added (iva) and import_export are positively associated with GDP growth, while new COVID-19 cases and government expenditure exhibit negative coefficients, consistent with pandemic shocks and potential crowding-out effects in the short run.

In terms of model fit, the R-squared values progress improve across Table 3: 0.078 (pooled OLS), 0.154 (FE-IV), 0.177 (FE), and 0.096 (PCSE-PSAR1). The substantial increase in R-squared from the OLS to FE and PCSE-PSAR1 models highlights the importance of accounting for province-specific heterogeneity and serial correlation in improving explanatory power. The FE-IV model has fewer observations (330) due to the use of lagged instruments.

The COVID-19 pandemic persisted for three years under continuous NPIs in China. China implemented a zero-covid policy to prevent the spread of the COVID-19 pandemic [6, 19], but these measures came with significant health and economic costs.^9^According to quarterly panel data, China's implementation of NPIs resulted in a 0.096 percentage point economic loss, a figure consistent with findings from [39] who estimated a 0.09(Table 5, column 1) economic impact across 44 countries. Similarly, Zachau [57] estimated an average treatment effect of −0.092, indicating that countries implementing a zero-COVID strategy achieved an average per capita GDP growth rate 0.092 percentage points lower than those adopting non-zero strategies. The economic toll was particularly severe in early 2020, when China faced its most stringent lockdowns. The closure of Wuhan, where 3,869 deaths^10^ were recorded, contributed to a 7.6% economic contraction in the region. Hubei^11^ Province's GDP plummeted by 19.3% in the first half of 2020,^12^while China's overall GDP fell by 6.8% year-on-year in Q1 2020.^13^This aligns with Barro et al. [8] who estimated a global GDP decline of 6% due to the pandemic. By 2022, many Chinese provinces were still enforcing NPIs, highlighting the prolonged economic and social trade-offs of these policies.

For regional dummy variables, in the PCSE_PSAR1 model, some provinces have coefficients with significance symbols, showing different economic impacts. Guangdong Province (coefficient = 3.508***) has a positive impact. This is mainly because Guangdong, as a strong economic province in China, has a large economic and a complete industrial system. Its manufacturing industry is developed, and industrial clusters such as electronic information, home appliances, and automobiles occupy an important position in the country and even globally, providing strong driving force for economic growth. It has strong scientific and technological innovation capabilities, attracting a large amount of talent and capital, and continuously promoting industrial upgrading and innovation development. In terms of foreign trade, as an important foreign trade window, it has many ports and frequent trade exchanges, providing strong support for economic growth.

Jiangsu’s marginal significance in GDP growth (p = 0.074) and its largest positive coefficient (4.421) in the PCSE, stem from its unique advantages in balancing pandemic prevention and economic development. Its precise, targeted NPIs avoided excessive shutdowns of core industrial chains (e.g., high-tech and equipment manufacturing), its solid industrial foundation and diversified economic structure enhanced resilience against pandemic shocks,^14^and its timely policy support and strong market vitality stabilized production and created new growth drivers, all of which effectively mitigated the negative impact of NPIs (the core independent variable) and supported positive GDP growth. Its marginal significance (rather than strict significance at the 5% or 1% level) is attributed to the overriding influence of common factors controlled by the PCSE, such as national economic downward pressure and the uniform impact of NPIs across provinces, which weakened the statistical robustness of Jiangsu’s province-specific positive impact.

Overall, the PCSE results consistently confirm that NPIs exerted a significantly negative effect on regional economic growth, while substantial heterogeneity exists across provinces depending on economic structure, industrial resilience, and policy effectiveness.

### Heterogeneity across areas and heterogeneity by year

To study the impact of the stringency of NPIs in China. According to the division of the National Bureau of Statistics of China,^15^ the province in China were categorized into three regions such as eastern provinces, central provinces, and western provinces. Given the prolonged duration of COVID-19, temporal heterogeneity was also analyzed to further examine variations over time (Table 4). Since provincial differences were already assessed in the previous analysis, the regression results for provinces are not presented in the heterogeneity analysis.

**Table 4:** Heterogeneity across areas and heterogeneity by year

	(1)	(2)	(3)	(4)	(5)	(6)
	area_east	area_middle	area_west	Y2020	Y2021	Y2022
NPI	−0.092***	−0.027	−0.092	−0.180***	−0.034	−0.012
	[−4.55]	[−0.62]	[−1.60]	[−3.42]	[−1.11]	[−0.49]
iva	0.219***	0.564***	0.251*	0.204***	0.408***	0.087**
	[3.42]	[13.95]	[1.92]	[4.21]	[4.01]	[2.07]
new_cases	0.000	−0.000	−0.000	−0.001***	0.001	−0.000**
	[0.31]	[−1.86]	[−0.96]	[−6.69]	[0.52]	[−2.29]
InGexpenditure	0.231	0.112	−0.057	1.598***	−1.106*	0.013
	[0.64]	[0.23]	[−0.29]	[3.12]	[−1.76]	[0.04]
import_export	0.218***	0.156**	0.083***	−0.050**	0.032	0.012
	[3.49]	[3.34]	[4.01]	[−2.09]	[1.63]	[0.73]
_cons	3.590	−1.002	7.179	−5.704	17.389***	3.746
	[1.75]	[−0.18]	[1.57]	[−1.00]	[2.96]	[1.08]
N	132	96	132	120	120	120
r2_a	0.576	0.743	0.359	0.749	0.601	0.344
F	167.731	646.285	29.326	1214.719	26.655	6.071

The regression results reveal notable regional and temporal variations in the impact of NPIs. Regional differences show that NPIs had a significantly negative effect across all regions, with coefficients of −0.0920*** (eastern), −0.027 (central), and −0.092 (western). The slightly stronger effect in the eastern region compared to the central region, along with the weakest impact in the western region, suggests regional disparities in NPI effectiveness. However, the smaller sample size in the central region (96 observations, compared to 132 in the east and west) may affect the robustness of these estimates.

Temporal heterogeneity indicates that NPIs had a significant negative effect in 2020 (−0.180***), but an insignificant negative effect in 2021 (0.034) and 2022(0.012). This weakening of NPI impact in 2021 could reflect policy adjustments or behavioral adaptations over time. Notably, the sample sizes for yearly regressions were balanced (120 observations per year), enhancing the reliability of temporal comparisons.

The temporal heterogeneity result (Table 4) provides clear evidence for a three-stage targeted response of NPIs on the Chinese economy and reveal its distinctive explanatory power over static models.

1. Initial phase (2020), stringent NPIs led to a significant economic contraction (coef. = −0.180, p < 0.01), establishing the steep initial trade-off predicted by the framework. This was primarily driven by severe supply chain disruptions and a collapse in consumption under uniform, high-stringency policies.
2. Mid-phase (2021) saw a statistically insignificant effect (coef. = 0.034). This pivotal finding aligns with the framework’s core mechanism of adaptation. It indicating that economic adaptation (e.g., shift to digital platforms, remote work) and substantial policy stimulus largely offset the costs of ongoing restrictions, effectively flattening the health-economy trade-off curve, a dynamic that static cost models cannot capture.
3. Post-vaccine phase (2022), NPIs exerted an insignificant negative impact (coef. = −0.012). This uncovers the later-stage dynamic of structural fatigue with the framework. The impact was likely due to the compounded effects of recurring Omicron outbreaks(The Economist, Apr 9th 2022), weakening policy effectiveness, and deteriorating external economic environments. This non-linear trajectory underscores that the public health-economy trade-off is not static but evolves dynamically over the course of a pandemic.

This non-monotonic trajectory of the estimated coefficients (significantly negative in 2020, insignificant in 2021 and 2022) aligns with and empirically observed our proposed three-stage theoretical framework of ‘Shock, Balance, and Adaptation’. It underscores that the public health–economy trade-off is not static but evolves dynamically through predictable phases over the course of a prolonged pandemic.

### Robustness checks

To ensure the reliability and robustness of our baseline findings, we implement a battery of sensitivity tests addressing concerns related to extreme policy shocks, dynamic policy effects, model misspecification, and outcome measurement. All robustness specifications maintain the identical control set and fixed-effect structure as the baseline model to ensure comparability, including industrial value-added growth (iva), quarterly new COVID-19 cases (new_cases), government expenditure (lnGexpenditure), import and export growth (import_export), as well as province and time fixed effects.

First, we exclude samples with extremely stringent NPIs to rule out bias driven by outliers. We define extreme lockdowns as observations where the NPI stringency index exceeds the 90th percentile. By dropping these top 10% of restrictive episodes, we test whether our core results are driven by a small number of harsh lockdowns.

Second, we use the one-period lag of NPI (L.NPI) as an alternative measure of policy stringency. This design accounts for the potential delayed effect of NPIs on economic activity and further alleviates contemporaneous endogeneity concerns, allowing us to capture lagged policy impacts.

Third, we replace the dependent variable with tertiary industry growth to test sectoral heterogeneity. Since contact-intensive service sectors are more vulnerable to mobility restrictions and gathering bans, using tertiary industry growth serves as a stricter test of NPI impacts.

Fourth, we adopt alternative inference methods better suited to short panels: Driscoll–Kraay standard errors, fixed-effects bootstrap inference, and feasible generalized least squares (FGLS). These approaches address the asymptotic limitations of PCSE when T < N and cross-sectional dependence is present.

As presented in Table 5, the coefficient of NPI remains negative and statistically significant across all robustness checks. In the baseline model, the NPI coefficient is − 0.096 and significant at the 5% level. When excluding extreme lockdown episodes, the NPI coefficient becomes − 0.153 and significant at the 1% level. Using the lagged NPI yields a coefficient of − 0.142, which is also highly significant at the 1% level. In the specification including both contemporaneous and lagged NPI, the contemporaneous coefficient is − 0.074 and significant at the 5% level. Finally, when employing tertiary industry growth as the dependent variable, the NPI coefficient is − 0.084 and significant at the 5% level.

**Table 5:** Baseline PCSE results and robustness checks

Variables	(1) PCSE	(2) Excl. Extreme	(3) Lag NPI	(4) Cont + Lag	(5) Tertiary	(6) Driscoll–Kraay	(7)FE–Bootstrap	(8) FGLS
NPI	–0.096** (0.046)	–0.153*** (0.014)	–0.078* (0.042)	–0.074** (0.038)	–0.084** (0.041)	–0.085** (0.033)	–0.002 (0.023)	–0.009 (0.009)
L.NPI			–0.142*** (0.040)	–0.161*** (0.039)				
iva	0.359*** (0.050)	0.367*** (0.042)	0.308*** (0.056)	0.307*** (0.055)	0.253*** (0.042)	0.387*** (0.107)	0.240** (0.113)	0.131*** (0.017)
new_cases	–0.000 (0.000)	–0.000*** (0.000)	–0.000 (0.000)	–0.000 (0.000)	–0.000 (0.000)	–0.000 (0.000)	–0.000** (0.000)	–0.000*** (0.000)
InGexpenditure	–1.383 (0.850)	–1.792*** (0.161)	–2.143*** (0.723)	–2.219*** (0.723)	–1.363* (0.777)	–0.279 (1.764)	7.489* (3.846)	0.383* (0.216)
import_export	0.129*** (0.013)	0.115*** (0.006)	0.110*** (0.011)	0.106*** (0.011)	0.135*** (0.013)	0.133*** (0.021)	0.009 (0.016)	0.009* (0.005)
Observations	360	324	330	330	360	360	360	360
R-squared	0.671	0.702	0.668	0.674	0.622	0.564	0.852	–

Panel-corrected standard errors in parentheses. All models employ panel-specific AR(1) correction and include province fixed effects and time trend. Column (1): PCSE-PSAR1 approach. Column (2): Excluding top 10% extreme lockdown samples. Column (3): Using one-period lagged NPI. Column (4): Including both contemporaneous and lagged NPI. Column (5): Dependent variable is tertiary industry growth. Column (6): Driscoll-Kraay, Robust standard errors for cross-sectional dependence. Column (7): FE–Bootstrap, Fixed effects with bootstrap inference (1,000 replications). Column(8): FGLS, Feasible generalized least squares

*p* < 0.10, ** *p* < 0.05, *** *p* < 0.01

## Discussion

This study’s empirical findings confirm that China’s NPIs during the COVID-19 pandemic induced an estimated 0.096 percentage point economic loss, with significant spatiotemporal heterogeneity, stronger negative impacts in eastern (−0.092) provinces than in western and central provinces, and a three-stage targeted response (significant negative impacts in 2020 (−0.180)), and an insignificant effect in 2021 (0.034) and 2022 (−0.012).

These results confirm the evolvable trade-off between pandemic control and economic stability. This discussion proceeds logically: interpreting core results through the health-economic trade-off theory centered on the dynamic balance between public health and economic stability, linking findings to prior empirical studies, deriving policy implications from spatiotemporal heterogeneity, and clarifying limitations and future directions.

### Health-economic trade-off theory

The health-economic trade-off theory captures the inherent tension between safeguarding public health such as curbing virus transmission and reducing mortality and maintaining economic stability including sustaining production and ensuring growth during public health crises. Its core insight that these two objectives often require mutual compromise provides a critical framework. Unlike many countries that oscillated between loose and strict measures, Dynamic zero-covid created a unique setting to test how this trade-off evolves under sustained NPIs, addressing a gap in existing research.

The three-stage temporal pattern directly illustrates the dynamic nature of the health-economy trade-off, reflecting the government’s adaptive adjustments amid competing priorities. In 2020, the emergency control phase, faced with the high-lethality initial COVID-19 strain, public health protection took absolute priority. Stringent and uniform NPIs such as Wuhan’s lockdown effectively suppressed virus spread and avoided medical system collapse but halted large-scale productive activities, leading to acute economic contraction. This phase embodies the “short-term economic sacrifice for long-term public health security” trade-off, which was unavoidable given the crisis’s severity.

In 2021, the balanced adaptation phase, with improved pandemic control experience and policy optimization, the trade-off shifted toward balance. Targeted interventions including lockdowns limited to high-risk areas and industry-specific exemptions and economic adaptation such as remote work, digital commerce and resumption of production mitigated NPIs’ marginal costs. Supported by policy stimulus, the economic impact of NPIs became insignificant, demonstrating that dynamic calibration can flatten the health-economy trade-off curve [23]. This is the core “dynamic balance” identified by the study. In 2022, the post-vaccine phase, the Omicron variant’s lower lethality, coupled with waning policy stimulus and deteriorating external economic conditions, disrupted the previous balance. NPIs were no longer proportionate to public health gains, and recurring outbreaks led to renewed significant economic losses. This phase highlights that the trade-off’s optimal point shifts with changes in pandemic characteristics and macroeconomic environments, requiring continuous policy adaptation.

Spatially, the stronger negative impacts of NPIs in eastern and central provinces compared to western provinces reflect regional differences in economic structure and sensitivity to the health-economy trade-off. This aligns with the health-Economic Trade-off Theory’s prediction that economic complexity amplifies policy impact. Eastern and central provinces such as Guangdong and Jiangsu have diversified economies dominated by manufacturing and services, relying heavily on smooth supply chains, labor mobility, and external demand. NPIs such as mobility restrictions and workplace closures directly disrupted these interdependent systems, intensifying economic losses. In contrast, western provinces’ economies are dominated by resource-based industries [44] and agriculture, which are less dependent on population mobility and complex inter-regional cooperation. Lower population mobility also reduced virus transmission risks, allowing for more flexible NPIs and mitigating economic impacts. This spatial heterogeneity confirms that the health-economy trade-off is not uniform across regions. Local economic structures must be considered to optimize policy design, avoiding a “one-size-fits-all” approach [12] that exacerbates regional disparities.

### Policy implications

Drawing on the [49] policy evaluation framework and this study’s empirical insights, a three-stage targeted response is proposed to minimize economic disruption while safeguarding public health during sudden outbreaks:

1. Initial phase (2020): the emergency control phase

Prioritized public health over economic concerns, with stringent and uniform NPIs (e.g., Wuhan’s lockdown) to suppress virus transmission. This phase incurred significant economic contraction (NPI coefficient −0.096**) due to halted productive activities, but successfully bought time to build systemic resilience (e.g., expanding healthcare capacity, establishing epidemic prevention mechanisms), avoiding catastrophic health impacts.

2. Mid-phase (2021): the balanced adaptation phase

Introduced balanced strategies that combined public health protections with economic recovery efforts. Policy adjustments (e.g., targeted lockdowns for high-risk areas, industry-specific exemption policies) and economic adaptation (e.g., remote work, digital commerce) mitigated the marginal cost of NPIs, resulting in an insignificant economic impact. This phase demonstrated that dynamic calibration can flatten the health-economy trade-off curve.

3. Post-vaccine phase (2022)

Emphasized flexibility and adaptability in NPIs. Amid recurring Omicron outbreaks and a changed macro-environment (e.g., waning stimulus, external economic pressures), the impact of NPIs in 2022 became statistically insignificant. This insignificance suggests that as the pandemic evolved, factors such as widespread vaccination, accumulated experience in targeted pandemic governance, and economic agents’ ongoing adaptation had alleviated the marginal economic costs of control measures, such that they no longer exerted a significant negative effect on economic growth. This phase still highlights the need to align interventions with vaccine-induced changes in pandemic severity and economic resilience, minimizing ongoing disruptions while safeguarding vulnerable populations.

It reveals the "a three-stage targeted response" of long-term mandatory public health policies: policy effectiveness depends not only on intensity but also on phased alignment with pandemic characteristics (lethality, transmissibility) and economic structures (industrial resilience, regional disparities). China’s practice offers critical global insights, when confronting prolonged public health crises, a dynamic coordination mechanism linking "pandemic situation, policy intensity, and economic support" must be established instead. This approach maximizes the comprehensive benefits of "life safety plus economic stability."

### Research limitations and future directions

This study has limitations. In terms of data collection, it relies on data obtained from public databases. Although the data sources have a certain degree of authority, there may be data incompleteness or inaccuracy issues. For example, some economic data of certain provinces may have missing values or errors due to reasons such as statistical calibers and data reporting, which may have a certain impact on the accuracy of the research results. A potential limitation of this study is that certain control variables (e.g., accumulated fiscal expenditure and industrial value added) may embed inherent time trends, which could confound the estimation of core coefficients. To mitigate this concern, we employed the two-way fixed effects (TWFE) model was adopted as the baseline specification, where time fixed effects (captured by time dummy variables) explicitly absorb common temporal shocks and linear trends across provinces, including those embedded in control variables. This approach effectively isolates the net effect of NPI stringency by filtering out time-varying confounding factors shared by all cross-sectional units.

Future research can explore the heterogeneous economic impacts of specific NPI [48] types based on OxCGRT classification to provide targeted precision control evidence. Fiscal and financial policies can be incorporated as moderating variables to analyze their buffering mechanisms on the health-economy trade-off. The research scope can be expanded to the urban–rural level to investigate how NPIs affect urban–rural health and economic inequalities, exploring micro-transmission paths to support urban–rural balanced development.

## Conclusion

Our empirical analysis of the economic impacts of NPIs underscores that balancing public health protection and economic sustainability. First, using provincial-level panel data and a sequential estimation strategy (including PCSE and FE-IV to address endogeneity), we confirm that NPIs significantly reduced quarterly GDP growth, with an estimated 0.096 overall economic loss, a figure consistent with global studies but uniquely validated under China’s prolonged "Dynamic Zero-COVID" strategy. Second, the impacts exhibit notable spatiotemporal heterogeneity: eastern provinces suffered stronger negative effects than western and central provinces, reflecting regional differences in economic structure and policy sensitivity; temporally, we identify an empirically observed three-stage dynamic pattern (significant negative impacts in 2020, insignificant effect in 2021and 2022) driven by policy adjustments, economic adaptation, and changing pandemic characteristics.

These findings validate the practical relevance of "dynamic balance" between public health and economic goals. They confirm that adaptive, context-specific adjustments, tailored to regional economic structures (e.g., supporting manufacturing/service-dominated eastern provinces with flexible mobility policies) and pandemic dynamics (e.g., calibrating NPI intensity with virus lethality and vaccine coverage), are not only feasible but critical to mitigating economic costs.

Overall, countries should maintain flexibility when implementing comprehensive control measures. In future responses to similar crises, policymakers must find a more reasonable balance between public health and economic development to effectively control the pandemic while minimizing its negative economic impact.

## Data Availability

All the materials relevant to this study are presented in the manuscript and its supplements. In case readers need to access the cited emails, they can request them from the authors, and the authors will provide them upon request.

## Abbreviations

C1–C8: Containment and closure policy indicators in OxCGRT
FE: Fixed Effects
FE-IV: Fixed-Effects Instrumental Variable
FGLS: Feasible Generalized Least Squares
GDP: Gross Domestic Product
gdpgrow: Quarterly provincial GDP year-on-year growth rate
IVA: Industrial Value-Added growth rate
L.NPI: One-period lag of NPI stringency index
NPI: Non-Pharmaceutical Intervention
OxCGRT: Oxford COVID-19 Government Response Tracker
PCSE: Panel-Corrected Standard Errors
PVAR: Panel Vector Autoregression
WALS: Weighted Average Least Squares

## Footnotes

1 WHO has 194 member countries *Countries overview | World Health Organization*. https://www.who.int/countries

2 https://www.worldometers.info/coronavirus/#countries

3 https://ig.ft.com/coronavirus-lockdowns/

4 For detailed classifications and specific connotations of non-pharmaceutical interventions (NPIs), including containment and closure measures (e.g., school closing, workplace closing, travel restrictions) and health system measures (e.g., public information campaign, testing policy, contact tracing), refer to supplementary appendix A.

5 The dataset downloaded from the China Bureau of Statistics has 31 provinces, with data missing for Tibet.

6 Tracking data on COVID-19, summary:*covid19 policy trackers*. https://lukaslehner.github.io/covid19policytrackers/

7 The official GDP growth rate of -39.2% for Hubei Province in 2020Q1 is sourced from Hubei Provincial Bureau of Statistics, "Quarterly Economic Operation Bulletin of Hubei Province (2020Q1)" [in Chinese], available at: https://tjj.hubei.gov.cn/tjsj/tjfx/qstjfx/202004/t20200421_2236918.shtml(accessed on 20 January 2025). The minor discrepancy between the official -39.2% and the study’s -39.3% stems from uniform decimal rounding for descriptive statistical consistency.

8 Since all variables are stationary in levels, cointegration tests, FMOLS, DOLS, and VECM are unnecessary and not applied in this study.

9 China's statistics bureau shows health costs 2020–2022 combined are 23 trillion-yuan https://data.stats.gov.cn/easyquery.htm?cn=C01

10 http://www.nhc.gov.cn/xcs/yqtb/202004/51706a79b1af4349b99264420f2cee54.shtml

11 Hubei Province, the worst affected area in China by the Covid-19, was declared under lockdown from 23 January to 8 April 2020 to prevent the spread of the virus.

12 https://www.ndrc.gov.cn/fggz/dqzx/dkzyyhz/202008/t20200826_1236853.html

13 http://www.mofcom.gov.cn/article/i/jyjl/e/202004/20200402956310.shtml

14 General Office of Jiangsu Provincial People’s Government. (2022). Implementation opinions on further unlocking consumption potential and promoting the recovery and high-quality development of consumption. Jiangsu Provincial Government Gazette, Su Zheng Ban Fa [2022] No. 50. Retrieved from http://www.jiangsu.gov.cn/art/2022/7/20/art_64797_10547127.html

15 https://data.stats.gov.cn/english/easyquery.htm?cn=E0102
